# Glycated Beef Protein Hydrolysates as Sources of Bitter Taste Modifiers

**DOI:** 10.3390/nu11092166

**Published:** 2019-09-10

**Authors:** Chunlei Zhang, Adeola M. Alashi, Nisha Singh, Prashen Chelikani, Rotimi E. Aluko

**Affiliations:** 1 Department of Food and Human Nutritional Sciences, University of Manitoba, Winnipeg, MB R3T 2N2, Canada; 2 Manitoba Chemosensory Biology Research Group, Department of Oral Biology, University of Manitoba, Children’s Hospital Research Institute of Manitoba (CHRIM), Winnipeg, MB R3E 0W4, Canada

**Keywords:** beef, protein hydrolysate, advanced glycation end products, Maillard reaction, electronic tongue, bitterness intensity, quinine, T2R4

## Abstract

Being averse to bitter taste is a common phenomenon for humans and other animals, which requires the pharmaceutical and food industries to source compounds that can block bitterness intensity and increase consumer acceptability. In this work, beef protein alcalase hydrolysates (BPAH) and chymotrypsin hydrolysates (BPCH) were reacted with glucose to initiate Maillard reactions that led to the formation of glycated or advanced glycation end products (AGEs), BPAH-AGEs and BPCH-AGEs, respectively. The degree of glycation was higher for the BPAH-AGEs (47–55%) than the BPCH-AGEs (30–38%). Analysis by an electronic tongue instrument showed that BPAH-AGEs and BPCH-AGEs had bitterness scores that were significantly (*p* < 0.05) less than quinine. The addition of BPAH-AGEs or BPCH-AGEs to quinine led to significant (*p* < 0.05) reductions (up to 38%) in bitterness intensity of quinine. The use of 3% hydrolysate to react with glucose yielded glycated peptides with a stronger ability to reduce quinine bitterness than when 1% was used. Calcium release from HEK293T cells stably expressing the T2R4 human bitter taste receptor was significantly (*p* < 0.05) attenuated by BPAH-AGEs (up to 96%) and BPCH-AGEs (up to 92%) when compared to the BPAH (62%) and BPCH (3%) or quinine (0%). We concluded that BPAH-AGEs and BPCH-AGEs may be used as bitter taste blockers to formulate better tasting foods.

## 1. Introduction

After the discovery of 25 bitter taste receptors and intensive efforts to elucidate the activation mechanisms of these receptors, the search for bitter taste modulators that can specifically block bitter taste receptors has gained increasing attention. So far, there are a few bitter taste inhibitors (γ-aminobutryic acid, Nα,Nα-bis(carboxymethyl)-L-lysine, homoeriodictyol and 4-(2,2,3-trimethylcyclopentyl)butanoic acid, probenecid, 6-Methoxylflavanones) that have been reported to act through interactions with bitter taste receptors [1,2,3,4,5,6]. Among them, N, N-bis(carboxymethyl)-L-lysine (BCML) is a derivative of the advanced glycation end product (AGE) called carboxymethyl-lysine (CML), which inhibits quinine-dependent activation of bitter taste receptor 4 (T2R4) with an IC_50_ value of 59 ± 18 nM [2]. Interestingly, CML inhibited quinine-activated T2R4 with an IC_50_ of 32.62 ± 9.5 µM; in contrast, it activated T2R20 with an EC_50_ of 65.31 ± 17.79 µM [7].

AGEs are a heterogeneous group of compounds generated from non-enzymatic reactions between the carbonyl group of a reducing sugar and an amino group of a protein, which is termed the Maillard reaction. The reaction process includes three stages, which are initiation, propagation and an advanced stage. The early glycation and oxidation processes form Schiff bases and Amadori products while further glycation of amino groups of peptides or proteins results in molecular rearrangements that lead to the generation of AGEs. The types and yields of final products are attributed to the selected reducing sugar types, pH, temperature, and heating time [8]. The process of AGE formation leads to the production of reactive oxygen species (ROS), which are believed to be deleterious to human health and contribute to several chronic diseases, such as diabetes, cardiovascular disease, neurodegenerative disease, and chronic kidney disease (CKD) [9,10]. However, peptides from soy protein and milk protein after the Maillard reaction were shown to possess enhanced antioxidant activity [11,12].

In addition to the aforementioned effects, AGEs are also undoubtedly important substances for generating a unique aroma and taste for thermally processed foods. For example, 4-hydroxy-2 (or5)-ethyl-5(or2)-methyl-3(2H)-furanone, 2-hy-droxy-3-methyl-2-cyclopenten-1-one, and 3-hydroxyl-4,5-dimethyl-2(5H)-furanone were reported to enhance the sweetness perception of sugar [13,14]. Moreover, the bitter taste intensity of casein peptide-derived AGEs decreased after 3 h heating, compared to heating casein peptides alone for 12 h [15]. It was also reported that Maillard reaction products of soy protein hydrolysates exhibited strong caramel-like odor and had a notably weaker bitter taste [11]. Recently we showed two AGEs, glyoxal-derived lysine dimer (GOLD) and CML were ligands of the bitter taste receptors that caused either activation (T2R20) or inhibition (T2R4) [7].

Meat has multiple proteins [16] and enzymatic hydrolysis offers the possibility of generating a wide range of peptides that have a desirable function as modifiers of bitter taste [5]. Alcalase has been commonly used to hydrolyze proteins because of its broad substrate specificity [17]. Alcalase has been extensively reported to improve food flavor, including Chinese sausage, soy protein isolates, yellow tuna and lean beef [17,18,19,20]. Additionally, beef protein alcalase hydrolysates (BPAH) and chymotrypsin hydrolysates (BPCH) have been demonstrated to have bitter taste-suppressing ability with confirmed activity at the taste receptor level [21]. Based on the demonstrated weaker bitter taste of certain Maillard reaction products [11], we hypothesized that glycation of beef protein hydrolysates would generate compounds that could negatively modulate the normal functions of T2R4, one of the most studied human bitter taste receptors. Therefore, in this study, BPAH and BPCH were reacted with glucose to produce AGEs and this was followed by the determination of their effect on T2R4 in cell-based calcium mobilization assays.

## 2. Materials and Methods 

### 2.1. Materials 

Ground beef was bought from the local market (Safeway, Winnipeg, MB, Canada). D-glucose, chymotrypsin^®^ (from bovine pancreas, EC 3.4.21.1) and Alcalase^®^ (from fermentation of *Bacillus licheniformis*, EC 3.4.21.62) were purchased from Sigma-Aldrich (St. Louis, MO, USA). BPAH and BPCH were produced as previously described [21]. 

### 2.2. Maillard Reaction

The Maillard reaction was carried out based on the protocol reported by Dong et al. [15]. Previously prepared BPAH and BPCH at concentrations of 1% and 3% (w/v) were each mixed with 100, 200, 300, 400, or 500 mM D-glucose in phosphate buffer, pH 10. The mixtures were heated at 120 °C in an oven for 30 min followed by rapid cooling in ice water and then freeze drying. In order to extract water soluble AGEs, 2 g of dried glycated hydrolysates were mixed with 50 mL water and 50 mL ethanol for 2 h at room temperature with continuous stirring. The mixture was then centrifuged (3270 *g* at 4 °C) for 30 min and the resulting supernatant collected while the residue was used to repeat the extraction procedure. Finally, the supernatants were pooled and evaporated using the vacuum rotary evaporator maintained at a temperature range between 35 and 45 °C. The concentrated supernatant was filtered through a 0.2 μm disc to remove insoluble materials and then freeze dried.

### 2.3. Degree of Glycation (DG)

Based on previous reports [22,23], the O-phthalic aldehyde method was used to determine the contents of free amino groups (FAG) of the BPAH and BPCH and their AGEs. The DG was calculated according to the following equation:

$$DG\;\left(\%\right)=\frac{FAG\;of\;hydrolysates-FAG\;of\;AGEs}{FAG\;of\;hydrolysates}\times \;100$$

### 2.4. Estimation of Bitter Scores by Electronic Tongue

Diagnostic solutions including 0.1 M HCl, 0.1 M NaCl and 0.1 M monosodium glutamate (MSG) as well as the calibration solution (1 M HCl) were purchased from Alpha M.O.S (Toulouse, France). Known bitter score substances such as acetaminophen, caffeine monohydrate, quinine hydrochloride, leporamide hydrochloride and femotidine were purchased from MP Biomedicals (Solon, OH, USA). Each freeze-dried AGE sample was dissolved in distilled water to give 0.5, 1.0, 2.0, 5.0 and 10.0 mg/mL concentrations followed by filtration first through a 0.45 μm polytetrafluorethylene (PTFE) filter disc and then a 0.2 μm PTFE filter. Bitter scores of AGEs were evaluated using the Astree II e-Tongue system (Alpha M.O.S., Toulouse, France). This system is a completely automated taste analyzer equipped with seven sensors, BD, EB, JA, JG, KA, OA, and JE, based on the ChemFET technology (Chemical modified Field Effect Transistor) to analyze liquid samples [24]. Firstly, 0.01 M HCl was used to condition and calibrate the sensors and the reference electrode repeatedly until stable signals were obtained for all seven sensors with minimal or no noise and drift. Secondly, the diagnostic procedure was performed repeatedly, using 0.1 M HCl, NaCl, and MSG to ensure the sensors could identify distinctive tastes, until the discrimination index achieved at least 0.94 on a principle component analysis (PCA) map. Following this, the bitter scores of AGEs were detected using 5 mg/mL, which was the minimum amount of sample that provided the required chemical signal on the instrument. The bitter scores were then projected using the bitterness standard partial least square (PLS) regression model of the instrument. The PLS bitterness standard model was constructed from several bitter taste compounds (caffeine, quinine, prednisolone, paracetamol, loperamide and famotidine) with known bitter taste scores that were determined by human panelists [24]. The specific concentrations are shown in Table 1.

### 2.5. Determination of Calcium Mobilization

Determination of the ability of AGEs to activate or block T2R4 was carried out by measuring intracellular Ca^2+^ mobilization using a Fluo-4 NW calcium assay kit [21]. Stable transfected HEK293T cells expressing T2R4 and G-alpha 16/44 or HEK293T expressing only G-alpha 16/44 were used as experimental and control groups, respectively. A viable cell count was taken after 6–8 h of transfection, and approximately 1 × 10^5^ viable cells/well were plated in the 96-well clear bottom black-walled BD falcon biolux plates, which were then incubated at 37 °C in a CO_2_ incubator for 16 h. Following this, the culture medium was substituted with Fluo-4 NW dye (lyophilized dye in 10 mL of assay buffer and 100 µL 2.5 mM probenecid added to prevent dye leakage from the cytosol) for 40 min at 37 °C in the CO_2_ incubator and followed by 30 min incubation at room temperature. Calcium mobilization was measured after the addition of AGEs to the cells in the presence or absence of agonist quinine and relative fluorescence units (RFUs) using a Flexstation-3 microplate reader (Molecular Devices, CA, USA) at 525 nm were recorded, following 494 nm excitation. Data were collected from two independent experiments each done in triplicate and PRISM software version 6.0 (GraphPad Software, San Diego, CA, USA) was used to generate the graphs.

### 2.6. Statistical Analysis

Data analyses were performed using one-way analysis of variance (ANOVA) with an IBM SPSS Statistical package, version 20 (Armonk, NY, USA). Mean values were compared using the Duncan Multiple Range Test, and significant differences were accepted at *p* < 0.05.

## 3. Results

### 3.1. Degree of Glycation

The degree of glycation (DG) of AGEs is summarized in Table 2, and it shows that BPCH-AGEs had significantly (*p* < 0.05) less values than the BPAH-AGEs. The DG improved with the increase of D-glucose concentration; thus, the AGEs produced with 0.5 M D-glucose had significantly (*p* < 0.05) higher values than the other AGEs from lower glucose concentrations. The relationship between the hydrolysate concentration and the DG of the AGEs was not linear. For example, the 1% (w/v) hydrolysate AGEs did not always have a lower DG than those produced from 3%. Therefore, degree of glycation was more dependent on glucose concentration rather than protein hydrolysate concentration.

### 3.2. Prediction of Bitter Score from Electronic Tongue

**Figure 1:**
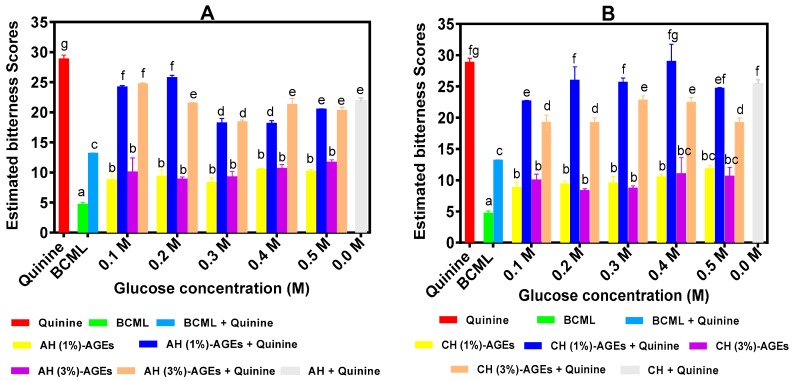
Estimated electronic tongue bitter scores of glycated protein hydrolysates and their ability to suppress quinine bitterness intensity: AH-AGEs, glycated alcalase hydrolysate (**A**); (**B**) CH-AGEs, glycated chymotrypsin hydrolysate (**B**). BCML (Nα, Nα-bis(carboxymethyl)-Llysine) was used as a positive control. Bars with different letters have significantly different (*p* < 0.05) mean values as determined from Duncan Multiple Range tests while error bars represent standard deviation.

Predicted bitter score of BPAH-AGEs and BPCH-AGEs are shown in Figure 1A,B, respectively. The results show that all AGEs (5 mg/mL) had significantly (*p* < 0.05) higher bitterness scores than that of BCML but significantly lower than that of quinine. Based on the acceptability of humans for bitter taste as shown in Table 3, the bitter taste of all AGEs (<15.0) is acceptable for human consumption. In the presence of BCML, quinine bitterness also significantly (*p* < 0.05) reduced. For a combination of BPAH-AGEs and quinine, AGEs from 0.3 M, 0.4 M, 0.5 M D-glucose concentrations combined with 1% BPAH had significantly (*p* < 0.05) lower bitter scores than other AGEs. In contrast, the effect of glucose concentrations on the reduction of quinine bitterness intensity by BPCH-AGEs was not significant. However, BPCH-AGEs prepared with a 3% hydrolysate concentration had significantly (*p* < 0.05) stronger suppression of quinine bitterness intensity than AGEs from 1%. 

The unglycosylated AH was more effective in reducing quinine bitterness when compared to some of the AGEs produced with 0.1 and 0.2 M glucose. However, the addition of 0.3 or 0.4 M glucose produced AGEs with a significantly higher suppression of quinine bitterness than the unglycosylated AH. In contrast, with the exception of the CH-AGEs from 0.1 M glucose, there were no significant differences in the quinine bitter taste suppressing ability of the AH-AGEs when compared to the unglycosylated CH.

### 3.3. Determination of Inhibitory Ability Against T2R4 Activated by Quinine

**Figure 2:**
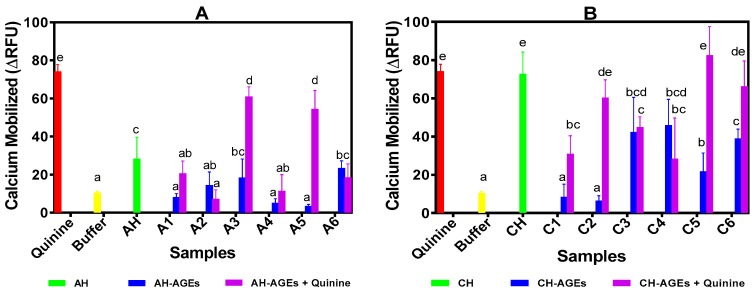
Calcium mobilization in T2R4 expressing HEK293T cell system after treatment with protein hydrolysates, glycated protein hydrolysates (5 mg/mL) and quinine (1 mM): **A.** alcalase hydrolysate (AH), 1% AH + 0.3 M D-glucose (A1), 3% AH + 0.3 M D-glucose (A2), 1% AH + 0.4 M D-glucose (A3), 3% AH + 0.4 M D-glucose (A4), 1% AH + 0.5 M D-glucose (A5), 3% AH + 0.5 M D-glucose (A6); **B.** chymotrypsin hydrolysate (CH), 1% CH + 0.1 M D-glucose (C1), 3% CH + 0.1 M D-glucose (C2), 3% CH + 0.2 M D-glucose (C3), 3% CH + 0.3 M D-glucose (C4), 3% CH + 0.4 M D-glucose (C5), 3% CH + 0.5 M D-glucose (C6). Bars with different letters have significantly different (*p* < 0.05) mean values as determined from Duncan Multiple Range tests while error bars represent standard deviation. ΔRFU: changes in relative fluorescence unit (test cells minus control cells).

In this study, HEK293T cells expressing T2R4-Ga16/44 were used as the experiment group, while HEK293 T cells expressing only Ga16/44 were used as the control group. Results of the calcium assay of AGEs are shown in Figure 2A,B for BPAH-AGEs and BPCH-AGEs, respectively. The results showed that all selected AGEs induced a significantly (*p* < 0.05) lower calcium release when compared to their respective hydrolysates, suggesting that the Maillard reaction reduced the bitter taste of beef protein enzymatic hydrolysates. Consistent with the electronic tongue data, calcium mobilization by BPAH-AGEs prepared with 3% hydrolysates was significantly lower than those of the 1% concentration. The BPAH-AGEs from 1% hydrolysate + 0.4 or 0.5 M glucose were especially found to be the least effective suppressors of quinine bitterness based on their significantly (*p* < 0.05) higher calcium releases when compared to other AGEs. The BPCH-AGEs also significantly (*p* < 0.05) decreased quinine-dependent calcium mobilization although this occurred with greater efficiency when prepared with 0.1–0.3 M glucose than 0.4 and 0.5 M glucose. 

## 4. Discussion

Glycation is a common method used to modify proteins, and the properties and characteristics of the resulting AGEs are closely related to the degree of glycation [25,26], which is influenced by many factors including temperature, time, water activity and the reactants molar ratio [12,27]. Previous studies reported that adding 500–1000 Da and 1000–5000 Da fractions of wheat gluten hydrolysates enhanced umami taste and improved the yield of AGEs [26,27,28]. However, cereals are rich in asparagines [29], which produce acrylamide, a known carcinogenic factor during the Maillard reaction [30]. Thus, meat protein hydrolysate is a better choice for producing safer AGEs. Given the same saccharide and same reacting conditions, the differences in DG could have been due to variations in the type of peptides within the enzymatic hydrolysates. BPCH had significantly (*p* < 0.05) lower DG than that of BPAH, suggesting that in addition to peptide size, the type and arrangement of amino acids on the peptides may be important in determining the degree of glycation. Li et al. [25] reported that the content of lysine and arginine decreased significantly in AGEs, implying that peptides with more lysine and arginine may have higher DG. In this case, we previously reported that BPAH and BPCH had similar contents of these two amino acids [21]. However, the higher DG of BPAH could be attributed to the presence of more of the short-chain peptides, which will mean a greater number of free amino groups available for the Maillard reaction. This is because our previous work showed that BPAH contained a higher level of short-chain peptides (average of 450 Da) when compared to the 1.8 kDa for BPCH [21]. Glycation is believed to be an important way of reducing bitter taste and generating aroma taste because the attachment of sugars can dramatically reduce the surface hydrophobicity of peptides and increase solubility [27,31]. Thus, the AGEs with higher DG may have a better taste and have higher potential for masking bitter taste.

Bitter scores of BPAH-AGEs and BPCH-AGEs were estimated by the electronic tongue. The ability of the AGEs to suppress quinine bitterness was stronger when 3% BPCH hydrolysate concentration was used but not BPAH. The greater bitter taste-suppressing activity of BPAH-AGEs from 0.3 M or 0.4 M glucose suggested the presence of complex glycosylated peptides with structures that promote better interactions with the T2R4 receptor when compared to the other AGEs. However, the BPCH-AGEs did not show a similar effect on glucose concentration. Therefore, it is possible that the peptide composition differed between BPAH and BPCH, which would explain the variation in the bitter taste-suppressing ability of the AGEs even when the same concentration of peptides was reacted with glucose. However, most importantly, the results suggest that these AGEs could serve as useful agents in blocking bitter sensation in the human mouth. The results are reliable indications of the potential use of the AGEs as bitter taste blockers. This is because the use of this instrumental approach was based on a previous report that showed the prediction of bitter taste of dairy protein hydrolysates had a R^2^ value of 0.94 when the electronic tongue data were plotted against the human sensory panel [32]. Although sensory tests are commonly performed by human panelists through physical tasting of samples, this method has some disadvantages, such as low objectivity and reproducibility, especially when large numbers of samples are involved [33,34]. However, human sensory tests have the advantage of representing the diverse taste preferences within the population, which cannot be measured by the electronic tongue instrument. The diversity in taste preferences and high variability may be useful for food product development that meets consumer demands. However, in order to solve some of the problems associated with human sensory tests, electronic-tongue instrumentation has been developed to enable the rapid analysis of a multiple number of samples within a short period of time and without the fatigue associated with human tasting. In the electronic tongue system, there is one Ag/AgCl electrode and seven sensors coated with lipid/polymer membranes, which govern the sensitivity and selectivity of an individual sensor by interacting with tastants to produce an electric potential [34,35]. This system can not only detect the bitter taste of samples, but can also determine the suppression ability of bitter taste modifiers, such as high potency sweeteners that suppress bitter taste of quinine hydrochloride, and acesulfame K and citric acid suppressing the bitter taste of epinephrine [36,37]. This study aimed to find highly efficient bitter taste modifiers, which were expected to be able to function at the taste receptor level. The results obtained in this work are similar to those we reported for various beef protein hydrolysates and peptides that also suppressed quinine bitterness intensity as measured by the electronic tongue [21]. In order to verify the results of bitter taste that were measured by the electronic tongue and further explore the mechanism of bitterness masking of AGEs, the AGEs with lower bitter taste scores were selected to conduct a calcium assay based on HEK 293 cells. Considering the diversity and complexity of 25 human bitter taste receptors, the T2R4 whose chemical structure and binding sites of its agonist quinine have been extensively studied, was selected as a future target receptor; quinine and BCML were selected as a bitter agonist and antagonist, respectively [2].

Based on the mechanism of the bitter taste transduction pathway, the activation of T2Rs will stimulate calcium release from intracellular stores, hence the calcium assay is used to identify bitter taste agonists and antagonists of T2Rs [2,38]. Furthermore, HEK293T cell-based heterologous expression is a robust assay method to measure G-protein coupled receptor activation and is wildly used for T2Rs and other human membrane proteins. Most of the T2R inhibitors including GABA, BCML, probenecid and 6-methoxyflavanones were discovered using the above HEK293T heterologous expression system and calcium assay method [1,2,3]. The results showed that some of the glycated peptides had a higher bitter taste blocking efficiency (reduced calcium release) than the hydrolysates. For example, glycated peptides like A1–A4 and A6 as well as C1, C3, and C4 had stronger effects in limiting calcium release than their corresponding non-glycated hydrolysates. For these samples, it is possible that glycation led to structural changes such as increased hydrophilicity, which reduced hydrophobic interactions with the T2R4 protein. In contrast, the AGEs with a weaker bitter taste blocking effect might have assumed structural changes that increased effective interactions (activation) with the T2R4 protein. The results are consistent with our previous report that showed a wide range of structural properties of beef protein-derived peptides that blocked quinine-dependent T2R4 activation [21]. However, we observed that there was no direct relationship between DG and quinine bitter taste-suppression, which suggested that the intrinsic structural properties of the AGEs may have had a stronger influence than simply the concentration of glucose used for the Maillard reaction. 

## 5. Conclusions

This study showed that the Maillard reaction efficiently reduced the bitterness of beef protein hydrolysates, which may be attributed to increased electrophilic properties of the glycated peptides. The increased electrophilic properties may be responsible for the decreased intrinsic bitterness of the glycated peptides and their enhanced ability to reduce quinine bitterness intensity as measured by the electronic tongue. The higher charged state of the glycated peptides could have reduced hydrophobic interactions with the T2R4 protein, hence the weak activation that was measured as a poor receptor response to quinine-induced calcium release (a measure of reduced bitterness intensity). The stronger ability of BPAH-AGEs to block quinine-dependent bitterness intensity may be due to the high state of glycation and poorer hydrophobic interactions with T2R4 (reduced receptor activation) when compared to the less glycated BPCH-AGEs. Future studies that are focused on structural elucidation of the AGEs will be required to determine the structural basis for the bitter taste-blocking ability. The use of human taste evaluation panels will be necessary to confirm the potential role of peptide AGEs as bitter taste-blocking ingredients in food and nutraceutical products development.

## Figures and Tables

**Table 1:** Compounds with known bitter scores from a human sensory analysis panel *.

Compounds	Used to Build Bitterness Standard Model	Used to Validate Bitterness Standard Model	Concentration (mM)	Published Values
Caffeine	√		0.24 2.36	2.5 8.5
Quinine	√		0.03 0.12	9 15.5
Prednisolone	√		0.44 0.88	13.5 17
Paracetamol	√		3.31 19.85	4 11
Loperamide		√	0.002 0.01	7.5 14
Famotidine		√	0.06 0.15	4.2 9

* Source: Alpha MOS [24].

**Table 2:** Degree of glycation (DG) for alcalase hydrolysate and chymotrypsin hydrolysate advanced glycation end products (AGEs).

Hydrolysate Concentration (%)	Glucose Concentration (M)	DG (%) *	
		Alcalase AGEs	Chymotrypsin AGEs
1	0.1	46.67 ^a^	29.53 ^a^
1	0.2	47.53 ^b^	29.11 ^a^
1	0.3	54.15 ^e^	30.13 ^b^
1	0.4	54.57 ^f^	36.67 ^d^
1	0.5	56.45 ^g^	37.14 ^e^
3	0.1	48.00 ^c^	29.49 ^a^
3	0.2	48.29 ^c^	28.86 ^a^
3	0.3	48.68 ^d^	36.63 ^cd^
3	0.4	54.88 ^f^	36.40 ^c^
3	0.5	54.96 ^fg^	38.60 ^f^

* Mean of triplicate replications. For each column, mean values with different alphabets are significantly different (*p* < 0.05).

**Table 3:** Bitter intensity level with corresponding scores from the human taste evaluation panelists *.

Intensity	Range
Taste not detected	1–4.5
Slight taste	4.5–8.5
Acceptable	8.5–12.5
Acceptable limit	12.5–16.5
Not acceptable	16.5–20.0

* Used to build the bitterness standard model [24].
